# Expression of p53 and p63 proteins and their correlation with histological grades among gastric carcinoma specimens at Mbarara Regional Referral Hospital

**DOI:** 10.4102/ajlm.v15i1.3000

**Published:** 2026-07-17

**Authors:** Lydia Jolly Ninsiima, Abraham Birungi, Richard Kasadha, Michael Junior Mugisa, Leuben Tibenderana, Nicholas Nuwashaba, Saphurah Nabaasa, Hassan Wasswa, Lawrence Amadile, Frank Ssedyabane

**Affiliations:** 1 Department of Medical Laboratory Science, Faculty of Medicine, Mbarara University of Science and Technology, Mbarara, Uganda; 2 Department of Pathology, Faculty of Medicine, Mbarara University of Science and Technology, Mbarara, Uganda; 3 Department of Medical Laboratory Technology, Faculty of Allied Health Sciences, Mayanja Memorial Medical Training Institute, Mbarara, Uganda

**Keywords:** gastric carcinoma, p63, p53, immunohistochemistry, histological grades, tissue

## Abstract

**Background:**

Gastric carcinoma remains one of the most common and deadly cancers worldwide, especially among older men. In sub-Saharan Africa and Uganda in particular, the diagnostic biomarkers of gastric carcinomas are not well documented; also, limited studies have been conducted in Uganda regarding the prognostic and diagnostic value of p53 and p63 immunohistochemical expression in gastric carcinomas.

**Objective:**

We aimed to determine the immunohistochemical expression of p53 and p63 proteins and their correlation with different histological grades among archived gastric carcinoma specimens at Mbarara Regional Referral Hospital.

**Methods:**

This was a laboratory-based cross-sectional study. We retrieved 166 archived formalin-fixed paraffin-embedded gastric carcinoma tissue specimens from the histopathology laboratory. They were stained with Harris haematoxylin and eosin stain and subsequently stained with p63 and p53 monoclonal antibody immunohistochemistry.

**Results:**

Of the 166 gastric carcinoma specimens retrieved, almost a third of specimens were poorly differentiated (53/166; 32%) although the majority were moderately differentiated (68/166; 41%). P53 expression was 20/45 (44.4%), 27/68 (39.7) and 21/53 (39.6) in well-differentiated, moderately differentiated and poorly differentiated gastric carcinoma. Statistically significant positive correlation was observed between p63 and weak immunohistochemical expression (Spearman rho 0.155, *p*-value 0.047).

**Conclusion:**

P53 expression was more than p63 expression in different histological grades. There was no significant correlation of p53 with different histological grades but significant correlation was observed between p63 and moderately differentiated gastric carcinoma specimens. We recommend a prospective study using advanced molecular tests on fresh gastric carcinoma specimens to assess the prognostic usefulness of p53 and p63 biomarkers.

**What this study adds:**

This study provides the first Ugandan data on p53 and p63 expression in gastric carcinoma, highlighting p63’s significant correlation with moderately differentiated tumours.

## Introduction

One of the most frequent and fatal malignancies worldwide is gastric carcinoma. It is the fifth most prevalent type of cancer, according to statistics.^1,2^ Similarly, the International Agency for Research on Cancers reports that gastric cancer is the second-most cause of cancer-related fatalities, followed by breast cancer, colorectal cancer, and oesophageal cancer.^3^ In 2025, in the United States, approximately 30 300 individuals (17 720 men and 12 580 women) were expected to be diagnosed with stomach cancer, and about 10 780 people (6400 men and 4380 women) to die from the disease.^4^ Incidence rates for gastric carcinoma in the United States rise with age, like other malignancies, and are uncommon in persons under the age of 50 years.^5^ Those over 50 years of age in the United States are most commonly diagnosed with gastric carcinoma.^6^

Africa has substantial regional heterogeneity, with estimated incidence rates ranging from 20.3/100 000 in Mali to 0.3/100 000 in Botswana.^7^ After Burundi, Uganda has the second-highest incidence and mortality rate of gastric carcinoma in East Africa (9/100 000, 8.7/100 000), with the Democratic Republic of the Congo having the lowest incidence and mortality rates at 7.3/100 000 and 7.2/100 000.^5^ Age-standardised incidence rates of gastric carcinoma decreased by 0.94% between the years 2001 and 2015.^5^ Research on gastric carcinomas conducted in Uganda at Mulago National Referral Hospital revealed that gastric carcinoma was common among tribes living in the volcanic regions of southwest Uganda.^8^

There is an over-expression of the p53 protein in a multitude of human malignancies, including gastric carcinoma and it is commonly accepted that the existence of p53 protein over-expression indicates the presence of a p53 gene mutation.^9^ Moreover, in cases of gastric carcinoma, the incidence and pattern of p53 over-expression may differ according to geographic origin.^10^ Tumour protein 63 (p63) is a transcription factor of the p53 gene family encoded by the TP63 gene located at chromosome 3q28.^11^ P63 has two major isotypes: TAp63 and black triangle Np63.^12^ P63 expression is found in basal cell layers of various organs, squamous epithelial cells of many organs including the gastric region.^12^ P63 expression was found in 101 gastric carcinoma tissue specimens and 25 normal gastric mucosa tissues by immunohistochemistry, according to Song et al.,^13^ who evaluated the expression of p63 in gastric tissue specimens and normal tissue specimens. Research demonstrated that elevated p63 expression in stomach specimens was significant when compared to normal tissue.^13^ Tumour protein 53 (p53) and tumour protein 63 (p63), two cancer biomarkers, may be utilised to forecast the course of gastric carcinoma.^12^ Nevertheless, the predictive usefulness of p53 gene mutations or p53 protein accumulation in gastric tumours and other types of human malignancy has not been thoroughly and consistently proven.^14^

The high mortality rate and poor prognosis suggest a poor understanding of the pathophysiology of gastric carcinoma cells. Cellular differentiation that takes place as a result of cancer progression can lead to p63 and p53 expression loss. These gene expressions are often missed since biomarker tests are not done and because patients present at an advanced stage of the disease.

The diagnostic biomarkers of gastric malignancies are not well understood in sub-Saharan Africa in general and in Uganda in particular. Immunohistochemical indicators are neither used routinely in the diagnosis or staging of gastric carcinoma nor used to track prognosis. Furthermore, there is no recorded study that has been done in Uganda discussing the prognostic and diagnostic relevance of p53 and p63 expression in gastric carcinomas.^15^ Thus, the purpose of this study is to determine the expression of p53 and p63 proteins in gastric carcinoma samples and their correlation with histological grades at Mbarara Regional Referral Hospital (MRRH).

## Methods

### Ethical considerations

Approval to conduct this research was sought from the Department of Medical Laboratory Science, Faculty Research Committee, Mbarara University of Science and Technology Research Ethical Committee (MUST-2022-426), and the administration of MRRH gave the administrative clearance. The archived gastric carcinoma specimens were de-identified and coded afresh to ensure the confidentiality of the persons from whom the specimens were obtained. After the study, the specimens’ blocks were re-archived to their respective areas. To avoid unwanted access, laboratory results were securely stored and shared only with those permitted. Also, we analysed archived samples which had been collected from patients who had given informed consent to have their samples archived and used for research purposes; this was in accordance with the declaration of Helsinki.

### Study design and site

This study was a laboratory-based, cross-sectional study conducted at MRRH in the pathology laboratory from 20 May 2022 to 15 September 2022. It involved collection of paraffin wax-embedded block tissues of patients diagnosed with gastric carcinoma. This laboratory serves as a national and regional histopathology laboratory in western Uganda. This laboratory is under the management and operation of Mbarara University of Science and Technology and located in Mbarara City, about 251 kilometres away from Kampala, the capital city. The laboratory receives about 23 gastric carcinoma specimens every year and mostly only relies on Harris haematoxylin and eosin (H&E) staining for all its diagnostic works.

### Study population

These were archived gastric carcinoma formalin-fixed, paraffin wax-embedded tissue blocks.

### Sampling procedure

Purposive sampling was performed by checking both histology record books and the laboratory information management system for only gastric carcinoma formalin fixed.

#### Selection criteria

All formalin-fixed, paraffin-embedded gastric carcinoma tissue blocks archived in the histopathology laboratory from 01 January 2017 to 04 March 2023 were included in the study. We excluded all tissue blocks of gastric carcinoma that were poorly processed and non-substantial for immunohistochemistry analysis.

#### Sample size calculation

The sample size was calculated using the formula for estimation of the single proportion.^16^ We therefore considered 176 specimens with 10% catered for specimens damaged during storage.

### Laboratory procedures

The laboratory procedures involved staining gastric tissue sections with H&E staining technique to confirm whether they were malignant (cancerous) and to categorise them in their respective histological grades. Later, those that were found to be malignant were stained using immunohistochemistry with specific monoclonal antibodies to p53 and p63, after which their staining intensity was scored using the immunoreactive scoring sheet of Remmele and Stegner. A score of 0 represented 0% staining and indicated no expression; 1 corresponded to < 10% staining with weak expression; 2 indicated 10% – 50% staining with moderate expression; 3 reflected 51% – 80% staining with strong expression; and 4 represented > 80% staining, also classified as strong expression.^17^

The tissue blocks were fixed on the microtome shunk (Thermo scientific Hm325) and cut into thin sections at a thickness of 5 microns using a disposable microtome knife. A rotary microtome (Thermo scientific Hm325) was used, and the tissue sections were put into a flotation water bath to straighten the wrinkles on the tissue section. Using a well-labelled slide, the tissue sections were scooped out perpendicularly. The water bath was set at 47°C, which was 10°C less than the melting point of the paraffin wax used. After sectioning, both sections for H&E and for immunohistochemistry staining were air dried at room temperature for 1 h. Finally, they were put in an oven at 52°C overnight before staining. The sections for immunohistochemical staining were placed on charged slides unlike the others for H&E staining.

#### Tissue staining with Harris H&E

The tissue sections were stained with H&E (in-house preparation) staining technique, which was a routine staining technique in the laboratory. The tissue sections were subjected to two changes of xylene for 6 min for deparaffinisation. The tissue sections were then brought down to water through different concentrations of alcohol (100%, 90%, 70%, 50%) for 15 dips in each container in a process called hydration, a process that allows the tissues to absorb haematoxylin, a water-based stain. The tissues were then subjected to haematoxylin stain.

**FIGURE 1:**
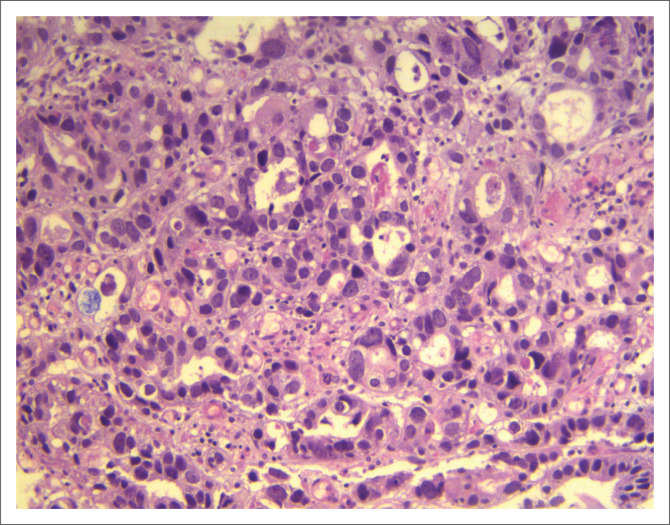
Well-differentiated gastric carcinoma tissue, haematoxylin and eosin, X 200, Southwestern Uganda, May 2022–September 2022.

The haematoxylin stain used was a progressive type called Harris’s haematoxylin stain, a basic dye that stains acidic elements in the cell (nucleus), which stained blue. The tissues were washed in water, blued for 5 min, and then counter-stained with eosin for 12 dips. Eosin is an acidic dye that stained the basic element in a cell (cytoplasm) pink. Histological preparations were reported according to grade as either well differentiated (Figure 1), moderately differentiated or poorly differentiated gastric carcinoma as used previously.^18^

#### Immunohistochemical staining

The sections mounted on charged slides were placed in the oven at 52°C overnight after which they were deparaffinised in three changes of xylene for 3 min each. They were hydrated to distilled water through descending grades of alcohol (i.e. 100%, 95%, 90%, 80%) then to distilled water. The sections were put into citrate retrieval buffer and put in the working chamber on top of a trivet. The bottom of the container holding the tissue was in good contact with water in the working chamber of the pressure cooker (Bio SB Tinto) retriever. The power cord was plugged into an outlet, and the MENU button was pressed on the control panel until the desired indicator light (high pressure of 110°C) was lit after which the TIME button was set for 15 min and the START button was pressed to start the procedure. As the pressure and temperature increased, the red pressure valve on top of the lid raised and stayed in place until the pressure was released. After that, the slides were rinsed three times in distilled water and they were circled carefully with a pap pen to contain fluids. The slides were placed in Tris-buffered saline (pH.7.6; Boston Bio Products, Inc.) and rinsed three times.

Then monoclonal mouse antihuman p53 antibody (clone DO-7) and p63 antibody (Biocare clone 4AF, mouse monoclonal) were each added to the gastric carcinoma sections separately and incubated for 1 h. After 10 min the gastric carcinoma sections were washed in distilled water, stained with Harris haematoxylin for 3 min, blued and then dehydrated in ascending grades of alcohol, cleared in xylene and mounted.

#### Tissue mounting

A drop of mounting media (D.P.X) was applied to the sections and the coverslip was carefully dropped onto the sample. If an air bubble was created in a mounted slide, it was removed by placing a sharp pointed object on it with gentle pressure.

#### P53 and p63 scoring procedure

**FIGURE 2:**
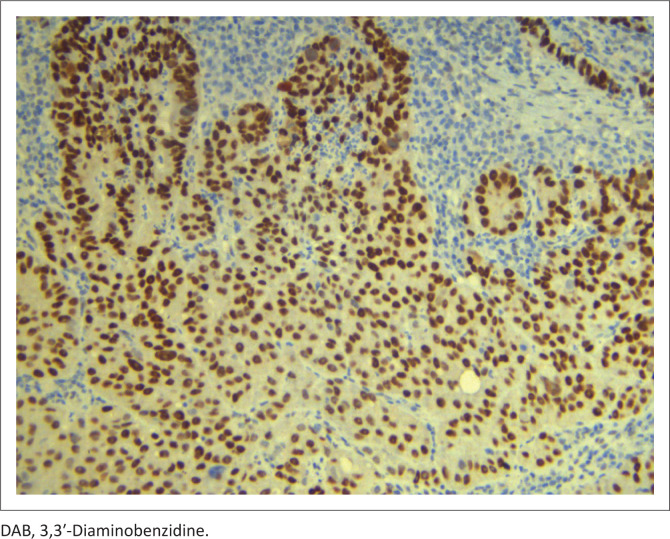
Gastric adenocarcinoma with positive reaction for P53. DAB, X 200, Southwestern Uganda, May 2022–September 2022.

**FIGURE 3:**
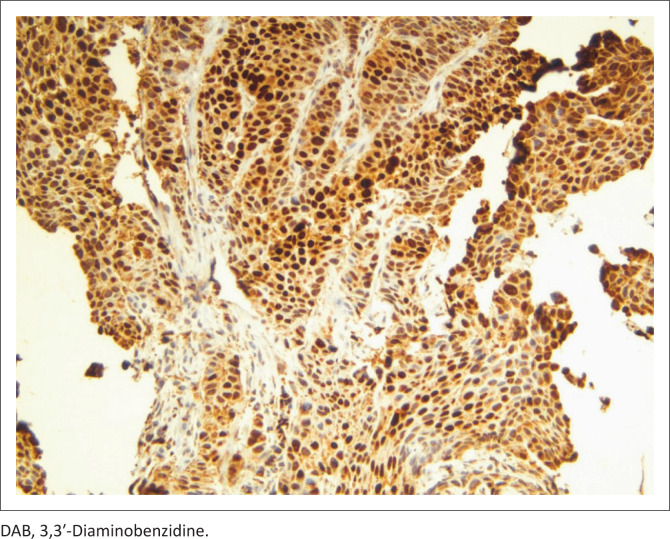
Gastric adenocarcinoma with positive reaction for P63. DAB, X 200, Southwestern Uganda, May 2022–September 2022.

In this study, we adopted a semi-quantitative grading system for p53 and p63 expression as used by Lazăr et al.^19^ The percentage of cells showing p53 and p63 expression was estimated using a 10X objective lens (Figure 2 and Figure 3);p53 and p63 expression were graded as negative if samples showed no positive reaction. When less than 25% of cells stained positive, the samples were graded as weak expression (+). When 26% – 50% of cells stained positive, the samples were graded as moderate expression (++) and when more than 50% of cells stained positive, the sample was graded as strong expression (+++).

### Data analysis

Data were entered, captured in an Excel sheet, cleaned, and entered into STATA version 17.0 software (Stata Corp., California, United States). The characteristics of specimens were described using proportions for categorical variables and mean, standard deviation and interquartile range for continuous variables. Tables and figures were used to represent the data elements. The expression of p53 and p63 among the gastric carcinoma was categorised as weak, moderate or strong. This was analysed using Chi square. To establish the correlation between expression of p53 and p63 proteins and different histological grades of gastric carcinoma, Spearman’s correlation was used. *P*-values of < 0.05 were considered statistically significant.

## Results

### Population demographic characteristics

The 166 gastric carcinoma specimens retrieved represent 166 source patients. Of them, 101/166 (60.8%) were from men, and mean age was 59.7 years (standard deviation 15.3). The majority of patients were above the age of 40 years (149/166; 89.8%). Almost half of the patients were residents of the greater Mbarara region (84/166; 50.6%). The median duration of specimen storage was 17 months (interquartile range: 9.5–28.8) as shown in Table 1.

**TABLE 1:** Demographic characteristics of retrieved gastric carcinoma specimens from the histopathology laboratory of Mbarara Regional Referral Hospital, Southwestern Uganda, May 2022 – September 2022.

Characteristic	*n*	%	Mean	s.d.	Median	IQR
**Sex of source patient**						
Female	65	39.2	-	-	-	-
Male	101	60.8	-	-	-	-
**Mean age of source patient in years (s.d.) Age category of patients in years**	-	-	59	15	-	-
< 40	17	10.2	-	-	-	-
> 40	149	89.8	-	-	-	-
**District of origin of patient**						
Greater Mbarara	84	50.6	-	-	-	-
Greater Bushenyi	29	17.5	-	-	-	-
Ntugamo	13	7.8	-	-	-	-
Kigezi region	22	13.3	-	-	-	-
Other	18	10.8	-	-	-	-
**Median duration of specimen storage in months**	-	-	-	-	17	9.5 – 28.8
**Categories of duration of specimen storage in years**						
< 2 years	108	65.1	-	-	-	-
≥ 2	58	35.0	-	-	-	-

s.d., standard deviation; IQR, interquartile range.

### Histological grades of gastric carcinoma at MRRH

Of the retrieved gastric carcinoma specimens, 45/166 (27.11%) were well-differentiated gastric carcinoma, 68/166 (40.96%) were moderately differentiated gastric carcinoma and 53/166 (31.93%) were poorly differentiated gastric carcinoma as shown in Table 2.

**TABLE 2:** The histological grades of gastric carcinoma specimens at Mbarara Regional Referral Hospital, Southwestern Uganda, May 2022 – September 2022.

Histological sub-grade	*n*	%
Well differentiated	45	27.11
Moderately differentiated	68	40.96
Poorly differentiated	53	31.93
**Total**	**166**	**100.00**

### Expression of p53 and p63 proteins in gastric carcinoma specimens at MRRH

Of all retrieved gastric carcinoma specimens, p53 was more expressed in moderately differentiated gastric carcinoma with 37/68 (54.4%), followed by well-differentiated gastric carcinoma with 20/45 (44.4%), and the least in poorly differentiated gastric carcinoma with 21/53 (39.6%). Out of 166 gastric carcinoma specimens, 6/166 (3.6%) stained weakly positive, 48/166 (43.6%) stained moderately positive and 24/166 (21.2%) stained strongly positive with p53 protein. Lastly, p63 was more expressed in moderately differentiated gastric carcinoma specimens with 10/68 (14.7%) followed by poorly differentiated gastric carcinoma specimens with 4/53 (7.6%) and lastly with well-differentiated gastric carcinoma specimens with 01/45 (2.2%). Out of 166 gastric carcinoma specimens 8/166 (4.8%) stained weakly positive with p63 protein, 7/166 (4.2%) stained moderately with p63 and the rest could not react with p63 protein as shown in Table 3.

**TABLE 3:** Immunoreactive score for expression of p53 and p63 proteins in gastric carcinoma specimens at Mbarara Regional Referral Hospital, Southwestern Uganda, May 2022 – September 2022.

Protein	Gastric carcinoma grade		Well differentiated (*N* = 45)		Moderately differentiated (*N* = 68)		Poorly differentiated (*N* = 53)		Test	*p*
			*n*	%	*n*	%	*n*	%		
p53	Weak expression	No	45	100	65	96	50	94	Fisher’s exact	0.33
		Yes	0	0	3	4	3	6	-	-
	Moderate expression	No	32	71	44	65	42	79	Chi-square	0.22
		Yes	13	29	24	35	11	21	-	-
	Strong expression	No	38	84	58	85	46	87	Fisher’s exact	0.96
		Yes	7	16	10	15	7	13	-	-
p63	Weak expression	No	45	100	62	91	51	96	Fisher’s exact	0.12
		Yes	0	0	6	9	2	4	-	-
	Moderate expression	No	44	98	64	94	51	96	Fisher’s exact	0.71
		Yes	1	2	4	6	2	4	-	-
	Strong expression	No	44	100	68	100	53	100	-	-

Note: All variables used are categorical. A chi square test was used on numbers that were five and above and those that were below five used a Fisher’s exact test.

### Correlation between expression of p53 and p63 proteins with different grades of gastric carcinoma at MRRH

There was no significant correlation between P53 expression and histological grades of gastric carcinoma. However, we observed a statistically significant and positive correlation (rho –0.155, *p* –0.047) between p63 weak expression with moderately differentiated gastric carcinoma as shown in Table 4.

**TABLE 4:** Correlation between expression of p53 and p63 proteins with different grades of gastric carcinoma specimen at Mbarara Regional Referral Hospital, Southwestern Uganda, May 2022 – September 2022.

Protein	Expression	Well-differentiated gastric carcinoma		Moderately differentiated gastric carcinoma		Poorly differentiated gastric carcinoma	
		Spearman’s rho	Prob. > *t*	Spearman’s rho	Prob. > *t*	Spearman’s rho	Prob. > *t*
p53	Weak expression	−0.115	0.141	0.037	0.658	0.072	0.361
	Moderate expression	−0.016	0.844	0.114	0.144	−0.106	0.178
	Strong expression	0.029	0.709	0.004	0.961	−0.031	0.689
p63	Weak expression	−0.134	0.086	0.155	0.047*	−0.031	0.636
	Moderate expression	−0.057	0.471	0.068	0.385	−0.019	0.812
	Strong expression	-	-	-	-	-	-

* , Significant values with *p*-value < 0.005.

## Discussion

In this study, immunohistochemical expression of p53 protein was shown in 78/166 (47%) of gastric carcinoma specimens. In contrast, various studies reported a lower expression of p53. For instance, Valente et al.^20^ in Japan reported that out of 66 gastric tissues tested, 25 (37.9%) had positive staining for the expression of the p53 protein. Also, a study conducted by De Souza-Pinto et al.^21^ reported that p53 protein was expressed in 41 of 163 (25.2%) surgical specimens of gastric cancer. This difference could be attributed to high-grade dysplasia in our specimens that expressed high p53 proteins. High-grade dysplasia is known to be associated with high expression of p53 protein.^22^

In addition, our study reported the expression of p53 protein in 78/166 (47%) of gastric carcinoma specimens which was lower than what was reported by Ando et al.^23^ who showed that out of 148 gastric tumours, 88 (59.5%) had p53 positive expression in gastric specimens This is could possibly be due to previous *H. pylori* infection that shows significant increase in proliferative activity and expression of p53 compared to uninfected individuals. Ando et al.^23^ recruited participants, the majority of whom had a previous *H. pylori* infection, which has been shown to increase proliferation in infected cells.^24^

Furthermore, our study results indicated that the expression of the p53 protein in gastric carcinoma specimens was similar to the findings of a study conducted in India by Hiralal Sankalecha et al.^25^ That study reported that 47.5% of their specimens showed p53 expression, which is closely similar with our findings of 47% (78/166 specimens). However, our results differed from those published by Hwang et al.,^26^ who observed p53 protein expression in 47.4% (37/78) of moderately differentiated gastric carcinomas. This discrepancy in findings could be attributed to the differences in staining procedures employed in our study compared to the automated staining platforms used by Hwang et al.^26^ for specimen processing. It is worth noting that automated staining methodology has been shown to offer higher sensitivity and reliability, as observed by Barreca et al.^27^

According to Lazăr et al.,^19^ the expression of p53 was observed in both moderately differentiated (47.4%) and poorly differentiated (29.4%) gastric carcinoma specimens in Romania. In contrast, our study found that the p53 expression in moderately differentiated and poorly differentiated gastric carcinoma specimens was rather higher (54.4% and 45.3%). The difference in our study could be attributed to the fact that we used only gastric carcinoma specimens, whereas Lazăr et al.^19^ used both benign gastric specimens and carcinoma specimens. Studies by Machlowska et al.^28^ and Zhang et al.^29^ support our findings, indicating that p53 is more expressed in gastric carcinoma than in benign gastric specimens.

Our study revealed that p53 is decreasingly expressed in moderately differentiated (54.4%), well-differentiated (44.4%), and poorly differentiated (26.4%) gastric carcinoma specimens. Furthermore, our study reported a p53 protein expression rate of 47% (78/166), while Busuttil et al.^30^ found a higher expression rate of 56.5% in gastric carcinoma specimens from Australia. These variations may be attributed to genetic differences between the populations of Australia and Uganda. Some studies have indicated that genes also play a role in inhibiting expression of some proteins.^31^

In this study, p63 expression was 9%, lower compared to the findings reported by Blanchet et al.^32^ from France, who demonstrated p63 expression of 40% in gastric tumours. Likewise, Tannapfel et al.^10^ reported a similar trend with a p63 protein expression in 22/46 gastric specimens (48%). The difference in expression may be attributed to the duration of specimen storage. It is possible that the stored tissues lose antigenicity, as illustrated by a study conducted by Ramos-Vara et al.^33^

In a study done by Steurer et al.,^12^ p63 expression was observed in 2.8% of studied tissues and was much lower than our findings which reported p63 expression in 9% of cases. The difference could be attributed to study population: in our study we recruited only gastric carcinoma specimens blocks while for the Steurer study, they recruited samples from different tumours, not gastric alone, and also included normal tissue in which p63 is not expressed. Song et al.^13^ conducted a study in China using 101 gastric cancer specimens and discovered a 48.5% (49/101) positivity rate for p63 expression. The discrepancy between the two studies could be attributed to differences in specimen type: the majority of their specimens were associated with metastasised lymph nodes, deeper invasion, and large tumour size, all of which contribute significantly to p63 positive expression.^12^

In the present study, p53 and p63 expression was more common in gastric carcinoma specimens that were moderately differentiated (54.4%), well differentiated (44.4%), and poorly differentiated (39.6%). This discovery is consistent with those made by Feng et al.,^1^ who found that well-differentiated gastric cancer expressed p53 more than moderately and poorly differentiated gastric carcinoma.

Gonçalves et al.^14^ conducted a study on p53 expression in gastric carcinoma and found that the expression of p53 was associated with histological grades and pathological grade (*p* < 0.05). This is consistent with a study conducted in India by Hiralal Sankalecha et al.^25^ In addition, our study observed a higher expression of p63 in poorly differentiated gastric carcinoma compared to well- and moderately differentiated gastric carcinoma (*p* < 0.05), which contrasts with our findings on p53 expression in well-differentiated gastric specimens (*p* > 0.005). We found similar results to the study by Song et al.^13^ It is widely recognised that excessive p63 protein expression is associated with a poor prognosis in various malignancies, as demonstrated by Lazăr et al.^19^ However, the correlation between p63 expression and clinicopathological characteristics in gastric carcinoma has not been thoroughly studied.^19^

A study that examined p63 expression in a small sample of gastric carcinomas found that few well-differentiated gastric carcinoma specimens exhibited higher p63 expression.^34^ This is contrary to the results of the current study. This difference could be attributed to the long archival duration (mean storage duration of 17 months) of the gastric carcinoma specimens used in our study. The length of amplifiable DNA and whole-genome-amplified fragments decrease with block storage, even with optimised DNA extraction procedures.^34^

A study done by Tannapfel et al.^10^ found that p63 was increased in gastric tumours (25/68; 37%) which is much higher than our study, which showed that p63 in gastric carcinoma is 9% (15/166). However, expression of p63 in our study indicated that there was a significant correlation of p63 with moderately differentiated gastric carcinoma specimens (rho = 0.155, *p* = 0.047). The difference in findings of the two studies could be attributed o differences in age. Studies have proven that gastric carcinoma expression is mostly expressed in old age.^35^

A study conducted by Wei et al.^3^ found that p53 is associated with variables such as gender, depth of invasion, lymph node metastasis, tumour necrosis factor, and lymphatic invasion but not with the grade of differentiation. According to a study by Steurer et al.,^12^ intense p63 expression (*p* > 0.05) was more commonly observed in cancers derived from p63-positive normal cell types, such as squamous cell carcinoma, regardless of their origin, than in histological grades.

A major strength of this study is that we used standard laboratory procedures as well as internationally acceptable scoring criteria for immunohistochemistry. This study was designed with the right statistical power to derive correlations. However, this study utilised archived gastric specimens in a histopathology laboratory. Loss of p53 and p63 could have occurred as a result of prolonged storage. Most of the gastric specimens used in this study were archived for more than a year. This study also relied on already collected demographic data on archived specimens. We were unable to study correlations between immunohistochemical expression of p53 and p63 with other clinical parameters including treatment outcomes.

### Limitations

This being an exploratory study, we only concentrated on two proteins (p53 and p63). We did not include other well-established markers including Ki67 and other mismatch repair proteins. Therefore, our study may not exactly represent the recommended comprehensive profiling of gastric carcinomas and may not be explicitly reproducible. This study utilised archived formalin-fixed, paraffin-embedded gastric carcinoma specimens, which may have experienced antigenicity loss owing to prolonged storage, potentially affecting p53 and p63 detection. The cross-sectional design relied on available demographic data only, limiting the ability to explore associations between protein expression and other clinical outcomes such as treatment response or survival. The study was conducted at a single referral hospital, which may restrict the generalisability of our findings to other populations in Uganda and sub-Saharan Africa. The use of immunohistochemistry alone without molecular techniques may not fully capture the mutational status or prognostic utility of p53 and p63.

### Conclusion

P53 expression in gastric carcinoma specimens was high while p63 expression in gastric carcinoma specimen was low. Both p53 and p63 were more expressed in moderately differentiated gastric carcinoma specimens. There was no significant correlation of p53 expression with different histological grades, but a significant correlation was observed between p63 and moderately differentiated gastric carcinoma specimens. The expression of p63 in gastric tumours makes p63 immunohistochemical staining a suitable diagnostic tool for gastric carcinoma. We recommend a prospective study on fresh gastric carcinoma specimens to assess the prognostic usefulness of p53 and p63 proteins while considering internationally recommended biomarkers including Ki67 and other mismatch repair proteins.
